# Understanding the healthcare provider response to sexual violence in Ghana: A situational analysis

**DOI:** 10.1371/journal.pone.0231644

**Published:** 2020-04-21

**Authors:** Lindsay M. Cannon, Emily C. Sheridan-Fulton, Roberta Dankyi, Abdul-Aziz Seidu, Sarah D. Compton, Amanda Odoi, Eugene K. M. Darteh, Michelle L. Munro-Kramer

**Affiliations:** 1 Department of Sociology, University of Wisconsin-Madison, Madison, Wisconsin, United States of America; 2 Research Consortium on Gender-Based Violence, Michigan State University, East Lansing, Michigan, United States of America; 3 College of Nursing, Michigan State University, East Lansing, Michigan, United States of America; 4 Department of Population and Health, University of Cape Coast, Cape Coast, Ghana; 5 Department of Obstetrics and Gynecology, University of Michigan, Ann Arbor, Michigan, United States of America; 6 Centre for Gender Research, Advocacy and Documentation (CEGRAD), University of Cape Coast, Cape Coast, Ghana; 7 School of Nursing, University of Michigan, Ann Arbor, Michigan, United States of America; Center for Primary Care and Public Health, SWITZERLAND

## Abstract

**Objectives:**

Gender-based violence is a global public health crisis, which has health, social, and economic impacts on survivors. In Ghana, responding to and preventing sexual violence on university campuses, has become a priority area. However, data are lacking on the healthcare provider response to students who have experienced sexual violence. The purpose of this study was to conduct a situational analysis to better understand the healthcare provider response to sexual violence in Cape Coast, Ghana.

**Methods:**

First, an observational facility assessment about healthcare services for survivors of sexual violence was conducted at two hospitals serving university students in Cape Coast, Ghana. Next, healthcare providers at the two hospitals completed: 1) a 113-item questionnaire about healthcare services, knowledge, and attitudes related to sexual violence and 2) in-depth semi-structured interviews describing their experiences providing healthcare to survivors of sexual violence. Descriptive statistics and frequencies were computed, and thematic analysis was used to analyze the qualitative data.

**Results:**

Both sites lacked supplies, including pre-packed rape kits, post-exposure HIV prophylaxis, and informational handouts on medications and support services for survivors. Further, healthcare providers lacked training on gender-based violence, including best practices for caring for survivors and evidence collection procedures. Providers described the clinical management for survivors of sexual violence, including providers’ role in reporting sexual violence to authorities, medical forensic exams, reproductive and sexual health services, and referral for mental healthcare. Finally, providers described a number of barriers to survivors accessing post-assault healthcare, including stigma and structural barriers, such as cost of medical supplies and lack of privacy within the healthcare facilities.

**Conclusions:**

The current healthcare response to sexual violence in Ghana is limited by lack of supplies, knowledge, and training for healthcare providers. Personal and structural barriers may prevent survivors from accessing needed healthcare following sexual violence.

## Introduction

Gender-based violence is a global public health crisis, occurring across geographic and cultural contexts. Gender-based violence, including sexual violence, is part of a broader societal problem in which gender inequality and social mores “normalize” violence and prime youth for later relationship violence [1,2]. Sexual violence can encompass unwanted sexual advances, unwanted sexual contact, and rape perpetrated by an intimate partner, friend, acquaintance, or stranger. Globally, it is estimated that 1 in 3 women will experience some form of physical or sexual violence by an intimate partner in her lifetime and one-third of adolescents report their first sexual encounter to be forced [3]. Despite the large number of victims in nearly every society, survivors of sexual violence often face numerous personal and structural barriers to accessing support and services [4].

Gender-based violence perpetrated by intimate and non-intimate partners results in significant acute and chronic health implications [3]. The consequences of gender-based violence include acute and chronic physical harm, psychological damage, increased use of health services after the assault(s), and impairment of personal relationships [5,6]. A survivor of sexual assault can experience a multitude of psychological reactions to the assault including anxiety, posttraumatic stress disorder, depression, eating and sleep disorders, substance abuse, suicide attempts, and sexual problems [3,5]. The outcomes of sexual violence often extend beyond the individual, impacting the family, the individual’s schooling and employment, and the economy. There is mounting evidence that trauma across the lifespan contributes to chronic disease [6], premature ageing, and ultimately, increased morbidity and premature mortality [7].

### Responding to sexual violence

Due to the pervasive nature of sexual violence, the World Health Organization developed a set of guidelines that outline the services needed by survivors of sexual assault [8]. These guidelines were created with the understanding that many countries are currently not meeting the needs of sexual assault survivors due to insufficient healthcare infrastructure. These guidelines recommend that the ideal facility would include: 1) an examination room with an examination table/couch, comfortable room temperature, privacy, clean bed linens, sufficient lighting, hand washing facilities, forensic supplies (either a rape kit or individual supplies like swabs, slides, blood tubes, etc.), an area to label specimens, lockable door, and telephone; 2) a separate waiting room where an advocate can talk to the patient; 3) shower and toilet for the patient; 4) room for the police; and 5) reception area for family and friends [8]. These guidelines also outline the medical management of patients with an emphasis on 1) male victims being triaged in the same manner as females, 2) universal procedures for consent, history, a head-to-toe physical exam focusing on injury, a genito-anal exam, and treatment; and 3) universal testing and treatment (for men and women) for sexually transmitted infections (STIs), including HIV [8]. The guidelines serve as a valuable resource to standardize protocols, improve quality, and educate the healthcare workforce.

### Sexual violence in Ghana

This situational analysis was conducted in Ghana, where responding to and preventing sexual violence on university campuses has become a priority area, although information about effective programs to prevent sexual violence is lacking. There is a culture of sexual violence on university campuses, which is undoubtedly linked to the high levels of sexual violence outside of universities [9–12]. According to a cross-country measure of discrimination against women in social institutions (i.e., formal and informal laws, social norms, and practices), Ghana scores as highly discriminatory [13,14]. Statistics indicate 27% of Ghanaian women have been sexually assaulted in their lifetime and for 20% of women, their first sexual experience is against their will [15]. According to the 2014 Ghana Demographic and Health Survey, gender-based violence is socially accepted, even among women, with nearly a third of women agreeing to at least one reason to beat your wife [16].

Accordingly, the government of Ghana has taken steps to safeguard citizens’ constitutionally protected rights to live free from violence. In 1998, the Ghana Police Service established a Women and Juvenile Unit that resulted in increased reports of violence against both women and children [12]. Additional action was taken in 2007, by passing Domestic Violence Act 732, which established a unit within the Ghana Police Service with the dual purpose of violence prevention and support of victims, and having laws which restrict access to and use of weapons [17,18]. Despite these legal provisions, socio-economic inequalities and gender norms in the socialization of males and females continue to create an atmosphere where violence is widespread, with women more likely to be the victims of violence [19].

These inequalities and gender norms inherently spill into the healthcare sector where healthcare providers are socialized within the same sociocultural context as individuals experiencing and perpetrating violence. Furthermore, healthcare providers often possess power and respect as part of their professional status that further imbalances their relationship with survivors of gender-based violence.

In 2007, the University of Cape Coast in Ghana approved a sexual harassment policy to protect and safeguard the rights of its students who have been or may experience sexual harassment (the term sexual harassment is used as an umbrella term to describe all forms of unwanted sexual contact–both verbal and physical). In addition, a sexual harassment committee was established to handle reported cases. The University of Cape Coast also established the Centre for Gender Research, Advocacy, and Documentation (CEGRAD) in 2013 to create a safe and inclusive space where women’s rights are protected. The sexual harassment policy was revised to include all members of the university community and guests who visit the university in 2015 and is now distributed as a handbook to all incoming first year students during orientation. However, there is a lack of structural interventions to ensure the healthcare infrastructure is equipped to respond to sexual violence.

In order to effectively avert sexual violence, prevention should occur at three levels, including primary prevention, or stopping the occurrence of violence, secondary prevention, or connecting survivors of violence to resources immediately following an assault, and tertiary prevention, which is focused on rehabilitation and long-term responses [20]. Ensuring students are receiving comprehensive healthcare in a trauma-informed manner is a secondary prevention strategy of paramount importance for survivors referred from the University of Cape Coast to a health facility. Trauma-informed care is an approach to healthcare which realizes the widespread impact of trauma and the various pathways to recovery, recognizes the signs and symptoms of trauma, responds in an integrated manner, and resists re-traumatizing patients [21]. However, one of the areas where information is lacking is in understanding the healthcare provider response to students who have experienced sexual violence in Ghana. Therefore, the purpose of this study was to conduct a situational analysis to explore healthcare providers’ practices and barriers to providing care to survivors of sexual violence in Cape Coast, Ghana.

## Materials and methods

### Design

We conducted an observational facility assessment, in addition to surveys and in-depth interviews with healthcare providers at two hospitals in Cape Coast, Ghana. The two sites are hospitals that provide healthcare to students at the university, as well as to members of the surrounding communities. This study was approved by the University of Michigan Health Sciences and Behavioral Sciences Institutional Review Board, the University of Cape Coast Institutional Review Board, and the Cape Coast Teaching Hospital Ethical Review Committee. We received comprehensive written informed consent from all study participants prior to the completion of any study activities.

### Procedures

First, a team of trained Ghanaian research assistants (a nursing student and a master’s of philosophy, population, and health student, both with research experience) completed observational facility assessments of supplies, protocols, and records related to sexual violence survivors at each hospital using the 58-item Sexual Violence Research Initiative (SVRI) Situation Analysis of Sexual Health Services for Survivors of Sexual Assault: Facility Checklist [22]. The checklist is based on the 2003 World Health Organization guidelines for victims of sexual violence [8] and assesses structural factors, medical and forensic supplies, protocols and information for patients, follow-up protocols, and healthcare records. This observational assessment helped to provide an overview of facility-level services and procedures [22].

Next, healthcare providers (including nurses, physicians, laboratory technicians, and mental health providers) working at either hospital were recruited via flyers posted in areas frequented by staff at each hospital (e.g., cafeteria, emergency room) and snowball sampling for surveys and in-depth interviews. Each flyer included information on the purpose of the study, the amount of time involved to participate, information about confidentiality, the incentive amount for the time involved, and contact information for the research assistants. Staff who specifically had experience in responding to survivors of sexual assault and those who work in areas that survivors would be most likely to access (i.e., the emergency department) were purposively sampled. For the healthcare provider survey, the 113-item SVRI Situation Analysis of Sexual Health Services for Survivors of Sexual Assault: Healthcare Providers questionnaire was completed [22]. First, providers were asked about their demographics, including their age, gender, position at the hospital, years working at the facility, and total years of experience. Next, providers answered a 63-item scale about providing care for survivors of sexual violence and a 27-item scale about training to provide care for survivors of sexual violence. Providers then answered 13 items on a sexual violence attitudes scale about endorsement of rape myths and attitudes related to providing care for survivors of sexual violence, measured from 1 (strongly disagree) to 4 (strongly agree). Eleven items were reverse coded and a sum score was created. Possible scores range from 13–52, with higher scores indicating more positive attitudes related to survivors of sexual violence. Sample items included, “It is disgraceful for women to bring rape cases to court,” and, “Only certain types of women are raped.” Finally, providers completed six items about knowledge of exams and multi-sectoral involvement.

Finally, the same healthcare providers participated in interviews led by the trained Ghanaian research assistants. Healthcare providers discussed their interactions with survivors of sexual violence, including how patients present to the healthcare center, history/exam procedures, and resources available before and after discharge. Sample interview questions included, “Do you care for survivors of sexual violence in your role as a healthcare provider?” and “Tell us what a healthcare encounter would look like for a survivor of sexual violence.” Interviews were digitally audio recorded and transcribed verbatim. Field notes were also used to capture significant interactions, body language, and quotes from the interviews. Healthcare providers received an incentive of GHC40 (approximately $10) for their time spent participating in the survey and interview.

### Analyses

All survey data were double-entered into SPSS (v.24). Descriptive statistics and frequencies were calculated. For the sexual violence attitudes scale, three participants had data missing at random. Due to the small sample size and small percentage of missing data on this scale (1.5%), these values were imputed using multiple imputation [23]. Sexual violence knowledge and attitudes of healthcare providers are presented using quotes from the semi-structured interviews. We also used the constant comparative method of analysis [24,25] and Dedoose (v.8.1.8) software to analyze the in-depth interviews to identify themes related to practices associated with caring for survivors of sexual violence, as well as barriers identified by these providers to seeking care faced by survivors. The study team used a systematic process to ensure the trustworthiness of the data. First, the first and second authors (LMC & ECSF) familiarized themselves with the data [26]. Then, four interviews were read and open-coded independently by these authors using an inductive approach to allow for themes to emerge organically [27]. The first and second authors (LMC & ECSF) then met to discuss their initial impressions and codes. A codebook of these inductively derived codes was created based on these four interviews and iteratively expanded as needed while coding the remaining interviews. All interviews were double coded. The first and second authors (LMC & ECSF) then met and sought concordance in the application of each code until all interview data were coded. These codes were reviewed and approved by all study authors.

## Results

### Study setting

Table 1 describes the results from the observational facility assessment in full. Both hospitals had a private room (defined as a room having four walls and a door) in which survivors of sexual assault could receive post-assault care. Additionally, both sites had HIV rapid testing and pregnancy testing available, as well as emergency contraception, analgesia, and antiemetics. However, neither site had pre-packed rape kits, emergency clothing, or post-exposure prophylaxis (PEP) for HIV prevention. Finally, neither site had informational handouts on medications, drug side effects, or support services for survivors of sexual assault.

**Table 1:** Results from the Facility Checklist measuring post-assault resources available at each site.

**Both sites**	Private room (with 4 walls and a door) for examination of survivors Examination gloves Sharps container Patient gowns Swabs Blood tubes Sheets of paper Pregnancy test kits in the examination room and available elsewhere in the facility HIV rapid test kit available elsewhere in the facility Emergency contraception available elsewhere in the hospital STI prophylaxis available elsewhere in the hospital Analgesia Tranquilizers Antiemetics Clinical management guidelines/protocols available in or nearby examination rooms Regimen for return visit HIV testing immediately post-assault and at 3- and 6-months Records kept of patients who have been examined for rape
**One site**	Private room available 24 hours Toilet in the same corridor as examination room Hot water in the healthcare facility Working telephone Speculum Colposcope Lockable cupboard for the storage of medical forensic evidence Lockable medical supply cabinet Sanitary towels Consent form for the medical forensic exam Paper bags HIV rapid test in the examination room Active follow-up after initial visit if the survivor does not return
**Neither site**	Bath or shower available to use after the examination Emergency clothing Pre-packed rape kits Post-exposure prophylaxis to prevent HIV Informational handouts on medications Informational handouts on drug side-effects Handouts on support services for rape survivors, such as NGOs

Missing data from one site on each of the following variables: 1) bed linens changed after each patient is examined (no at one site), 2) examination couch (no at one site), and 3) working angle lamp (yes at one site).

### Study sample

Table 2 describes the demographics of the healthcare providers who participated in both the survey and semi-structured interviews. Data were collected from 20 healthcare providers, including 7 doctors, 10 nurses, 2 laboratory technicians, and 1 mental health provider. Mean age was 30.6 years and 55% of the providers were female (n = 11). Providers had worked at their current site for an average of 3.8 years and had worked in their field for an average of 5.7 years. On the sexual violence attitudes scale, the mean score was 38.8, indicating neutral attitudes towards survivors of sexual violence. Interviews ranged from 11–41 minutes in length, with a mean of 20.3 minutes.

**Table 2:** Sample characteristics (N = 20).

	% (*n*)
Type of healthcare provider	
Doctor	35% (7)
Nurse	50% (10)
Laboratory Technician	10% (2)
Mental Health Provider	5% (1)
Mean age, years (range)	30.6 (25–41)
Sex	
Female	55% (11)
Male	45% (9)
Years of service at the site, years (range)	3.8 (0.25–10.0)
Total years of service, years (range)	5.7 (0.42–12.0)
Received formal training on the clinical management of survivors of rape	50% (10)
Sexual violence attitudes scale sum score, mean (range)	38.8 (32–48)

### Provider survey

Half of the providers (n = 10) had received formal training on the management of sexual assault. The most common form of training (n = 10) involved undergraduate course work during which care for sexual assault survivors was covered. Thirty percent (n = 6) had participated in an in-service training. Seventy percent of providers (n = 14) reported that they would like to receive additional training on working with sexual assault survivors. Forty percent of providers (n = 8) had seen at least one adult sexual assault survivor (range 0–10 survivors) presenting for services over the last three months. Similarly, 45% of providers (n = 9) had provided care to at least one child survivor of sexual assault (range 0–10 survivors) over the past three months.

Thirty percent of providers (n = 6) stated that most survivors of sexual assault they provide care for report to the police, while 10% (n = 2) said some survivors report, 55% (n = 11) said few survivors report, and one provider stated that they did not know. Seventy percent of providers (n = 14) reported that a survivor is not sent to the police station before a physical exam can be completed if they present to the healthcare facility prior to the police station. Further, 70% of providers (n = 14) reported that police are not routinely called to come to the health facility if someone presents at the healthcare facility first following sexual assault. Eighty percent of the providers (n = 16) stated they routinely recommend a full medical forensic exam to collect evidence, regardless of whether a survivor chooses to report to the police or not.

Related to post-assault healthcare concerns, 90% of healthcare providers (n = 18) reported that survivors of sexual assault express concerns related to HIV infection, 75% (n = 15) reported that survivors are concerned about STIs, and 85% of providers (n = 17) reported that survivors are concerned about pregnancy. Forty percent of healthcare providers (n = 8) reported that survivors express concerns related to mental health.

### Provider knowledge

Providers’ knowledge on sexual violence was assessed in the semi-structured interviews. Providers consistently described gender-based violence as violence inflicted upon a particular gender, most commonly women. However, some providers were not familiar with the term “gender-based violence,” as reflected in the following statement, “I have not heard of the terminology before…” (male doctor). Physical assault, such as being “beaten and stuff” (female nurse), was the form of violence that was most commonly reported; however, some providers also discussed sexual or psychological abuse as forms of gender-based violence. Most providers described sexual violence as a range of behaviors, including rape or non-consensual penetrative sex, verbal harassment, and other non-consensual forms of sexual contact. However, a few providers conflated rape and sexual violence, defining both as non-consensual penetrative sex. Provider knowledge related to coercion was more varied. Some providers described coercion as “basically forcing the person to do something against his or her will” (male doctor), while others discussed tricking, convincing, intimidating, or threatening someone to do something they do not wish to do.

Some of the providers noted that violence is fueled by abuses of power. For example, half of respondents discussed cases where power was abused to force sexual encounters through threats or physical force. Abuses of power were typically described as cases in which the abuser was older than the person who was abused, such as a teacher, an uncle, or a caretaker.

### Provider attitudes

Providers’ attitudes related to causes of sexual violence and justifications for violence were explored in the interviews. Participants described many causes of sexual violence including, but not limited to: a lack of self-control and over-active sex drive on the part of the perpetrator (n = 9), provocation by the survivor through seduction or the way they dressed (n = 6), substance use by the perpetrator (n = 4), poverty (n = 3), broken homes (n = 2), poor relationships (n = 2), and pornography (n = 2). Providers also discussed the ineffective prosecution of perpetrators as another potential cause for sexual violence. For example, one male laboratory technician said that the “punishment meted out on the perpetrators of sexual violence is not deterrent enough,” while another female mental health provider mentioned that “perpetrators know they can just go scot-free.”

When discussing justifications for violence, most providers (n = 18) agreed that there was no justification, whether it was the male partner being violent towards his female partner or vice versa. One female nurse said, “Nobody has any right.” A small number of providers (n = 2) gave examples of when violence would be acceptable. One female nurse said it would be justified “when the lady starts with or starts raining an insult on a man.” Another female nurse said, “There is no justification when it comes to sexual violence,” when referring to instances when the male partner was being violent, but she said it was acceptable for the female partner to be violent when seeking revenge for a previous incident when the male was violent.

### Interview themes

There were two themes that emerged from the qualitative data that adhered to the study purpose of exploring practices and barriers to providing care among healthcare providers. These two themes, clinical management and survivors’ barriers to seeking healthcare, illustrated providers’ experiences within the sociocultural context as well as specific elements derived from their clinical practice.

#### Clinical management

During the interviews, four topics related to the clinical management of care for survivors of sexual violence were described in-depth, including providers’ role in reporting sexual violence to authorities, the medical forensic examination, reproductive and sexual health services, and referral for mental healthcare.

***Providers’ role in reporting sexual violence to authorities***. During the interviews, providers often discussed their role in reporting to authorities, as well as the pathways through which survivors could arrive at a healthcare facility. There were three pathways for reporting the sexual assault to others. The first pathway described was to disclose to community elders and/or religious leaders. One male doctor described how community elders would occasionally step in to mediate between the survivor and the perpetrator when a survivor decided to keep the report within the family, stating, “So we have a wise person in the family who will sit the couple down and they will hash out that issue.” The second pathway described by healthcare providers involved survivors presenting to the hospital where they would receive healthcare first and then often be encouraged to go to the police. If the survivor went through the second pathway, they sometimes came to the hospital with a family member or friend. A number of providers also talked about including the family if they were present, such as encouraging the family member and survivor to report to the police or stating that elderly family members or parents were resources that a survivor could use to report. The third and final pathway described by the healthcare providers involved the survivor going to the police to make a report and then coming to the hospital to get the “police form” completed. This paper form is filled out by the healthcare provider–with information about the exam and testing performed–and returned to police. As noted by one male doctor, “In Ghana … it starts from the family and siblings, then to the churches and mosques, then to the police. They must report to the police so they will request for a medical exam to be done.” If this pathway was chosen, some providers believed that survivors only came to the hospital to get their police forms signed.

Nearly half of providers stated that they would encourage a survivor to report to the police, but ultimately it was a survivor’s choice whether or not to report. A few said that they always notify the police in cases of assault. One provider noted that a positive screen for an STI in a patient who is a child results in an immediate report of sexual violence to legal authorities. Providers raised several potential factors that influence survivors to not report their experiences to the police, including a desire to keep their experiences private, concern about not being believed, and avoidance due to stigma.

***The medical forensic examination***. Providers described the medical forensic examination procedures for patients who present for post-assault care. As one male physician described:

*During our first interaction with them*, *usually what we let them know is that everything they share with us will be confidential*. *We also assure them that we will try and keep their dignity intact so they shouldn’t be too worried about unnecessary exposure*, *which will not be of help*. *We initially take a thorough history to try and find out who the assailant was*, *if it was a known or unknown person*. *Was it a single person or it was multiple persons*? *We would also want to find out the nature of the sexual assault or the alleged sexual assault*, *whether [it] is penile/vaginal*, *oral*, *and/or any sort of sexual violence*. *And we also finally*, *after we have gotten the history*, *we do a thorough examination from head to toe*. *Not only do we zoom in [on] the genital area*, *but then we look for any signs of trauma from up*, *down*. *And then finally when we are done with that*, *we now do our genital examination*, *which sometimes may involve photography and then instrumentation*. *So*, *depending on whether we receive consent or not*, *we know how far we go*.

This description details the steps in the medical forensic exam, including a thorough report of the sexual assault, followed by an examination of the survivor’s body conducted by a provider with a chaperone present to assess for injuries and collect evidence. As many patients present to the hospital seeking the completion of a “police form,” most providers described the collection of evidence and completion of the legal form as major components of the medical forensic exam. As one female physician described, “Because it’s a legal issue, you try to deal with the legal aspect …So you try to find evidence that this has actually happened from your medical point of view.” Many providers reported that patients often present for medical forensic exams long after the sexual assault occurred, making evidence collection difficult. As one male laboratory technician reported, “Most of them come in late all the time so we don’t get any concrete evidence to punish the perpetrators of these victims.”

Most providers noted that survivors must give consent prior to the exam and have the right to decline any part. However, if survivors wish to have the police form completed as part of their report, they are unable to decline portions of the exam. As one female physician described, “And for that form, you actually need to document everything. So, a lot of the times they consent to it because that is the only proof you can get.” Further, a female nurse described survivors as lacking agency in deciding which parts of the exam they wish to complete. She stated:

*At the instance the survivor is at our mercy*. *We make the survivor feel like there is something we can do immediately to alleviate the pain that he or she is going through*, *so we just expect him or her to be following our instructions…‘Take off your clothes and lie down*. *I am saying it this way*, *are you not hearing what I am saying*?*’ And then*, *because the person is already in pain*, *he is forced to conform to whatever we are asking of him*.

Finally, several providers described “convincing” patients into completing portions of the exam. As one female nurse reported, “Normally what happen[s] is that if there is [an] exam and [a] certain lab that we would want that survivor to pass through, we let the survivor know the reason and the importance of her going through that lab. But if the patient refuses, it’s her right, but we make sure we convince her enough to make her go through it.”

Providers discussed a few challenges related to the medical forensic examination. First, providers described not having a “special unit that deals with rape.” Providers also discussed the lack of access to pre-packed rape kits, which were not available at either location. Multiple providers described the lack of rape kits as a systemic problem that can be observed across healthcare systems in Ghana. One male laboratory technician stated:

*I believe that in Ghana*, *the healthcare system is not serious about tackling the problem of sexual violence*. *As I’ve said previously*, *the most important thing to do once sexual violence is reported is to take sample[s] to scientifically ascertain sexual contact between the alleged perpetrator and the alleged victim*. *And as I said*, *that can only be done with a DNA test*. *So*, *if we are serious about nailing people who are alleged to have perpetrated sexual violence* …*there are simple sample collection kits that can be made available to healthcare institutions so that immediately it happens*.

One male doctor also discussed a lack of knowledge about what happens to collected evidence after the examination due to lack of storage, stating:

*And we kind of seal them and let them know that these things are for rape victims but genuinely we don’t know what is done with them after*. *Maybe whether when we request for them two months later whether they can be able to provide those samples* …*And then we are assured that there is no break in the chain of transport…But storage of stuff*, *no*, *underwear*, *shavings*, *and others*, *no*, *we don’t have anywhere that we keep those*.

Finally, multiple providers described a need for additional training on managing care for survivors of sexual violence, as many have never received any formal training or found the training that they had received to be inadequate for providing sensitive care. As one female nurse stated, “Such victims cannot be managed as the ‘normal’ ones. So that if we are taken through training, we will give them specialized care. We will not handle them as the ‘normal’ patient. And then we will also not do anything that will aggravate their pain because one word from a nurse can worsen the situation.” Beyond providing sensitive care, providers also noted that without training, they are unfamiliar with the correct protocols and procedures for performing medical forensic exams. One male laboratory technician stated, “I need education on how to handle samples from people like that to ascertain whether, in fact, this person has had sexual contact with this other person. We need scientific proof and I haven’t had any formal education on how to either, one, collect samples or, two, work on such samples.”

***Reproductive and sexual health services***. Providers described post-assault sexual and reproductive health service provision as a multi-step process. First, female survivors who present for services related to an assault are screened for pregnancy and all survivors are screened for STIs. However, as one provider noted, “It is not realistic at that point to do an HIV test or any of those other sexually transmitted infections,” as new infections would not show symptoms at the time of initial hospitalization if the assault had occurred recently. Further, at the initial visit, patients are provided with STI and pregnancy prophylaxis if they have the funds to pay for these medications. However, two physicians stated that if the survivor “knows the person’s status, like in terms of sexually transmitted infections,” then post-exposure prophylaxis is not provided to the survivor. One provider stated that survivors are also provided with education related to pregnancy and STI risk following an assault. Following the initial visit, female patients are scheduled to return for follow-up testing for pregnancy and all survivors are scheduled for return STI testing, including HIV testing, at 3-months post-assault.

Providers noted that survivors’ need for sexual and reproductive health services is often the catalyst for seeking post-assault services. In particular, multiple providers noted that one of the main reasons female survivors seek services is to access pregnancy prophylaxis or termination services following an unwanted sexual encounter. One female nurse stated, “Pregnancy, the fear of becoming pregnant might be one of the key thing[s] that will push an adult to come and report about rape or when pregnancy sets in, that’s when she will report. Even when she reports, it [is] about termination. She is coming because she wants to access a facility that can help her terminate the pregnancy, not because of any other reason.”

***Referral for mental healthcare***. Regarding the provision of mental healthcare, both hospitals had at least one counselor on site and the majority of healthcare providers described referral to mental health practitioners as an important step in post-assault care for sexual violence survivors. One male doctor stated, “So we schedule a visit for the patient to see the clinical psychologist where they will be taken through [a] series of counseling to help them cope with the trauma that they’ve gone through.” However, due to the small number of mental healthcare providers at each site, one male doctor discussed difficulty in connecting survivors of sexual violence with psychological services, stating:

*Even sometimes arranging meetings with psychological people like the psychologist to at least take them through some psychologic[al] counseling is quite difficult…I think once they come*, *we realize they are not pregnant*, *nobody really takes care of their psychosocial component of the rape…But we feel like there are other components of it that we don’t know how to handle*, *especially the psychological aspect of it*. *So*, *I think that is one area that we could take a closer look at*.

A female mental health provider described the range of mental health services provided to survivors of sexual violence, stating, “Get into the background, find out exactly what happened and then use assurances, empathy and the rest, cognitive restructuring that they can get on and all of that …Some of them need, they just need hope and encouragement…Sometimes they are afraid of their physical environment and they need to get over the trauma.” This mental health provider also described her role in advocating for young pregnant survivors to remain in school and helping survivors to manage interpersonal dynamics within their social support systems. Finally, a female mental health provider discussed her role as the main source of continuing follow-up for survivors, stating, “My clients, I meet them not less than five times just to get to know their adjusting and their coping.”

#### Survivors’ barriers to seeking healthcare

Providers noted barriers that they believe interfere with survivors’ ability to seek care following sexual violence, including stigma and structural barriers, such as cost of treatment and lack of privacy.

***Stigma***. Providers discussed the role of stigma as a barrier to survivors seeking health services following sexual violence. This stigma manifests in multiple ways, including lack of social support and pervasive silence. Providers discussed how survivors are encouraged to remain silent by family and community leaders who “try to shelve it” and “try to make it a home matter” (male laboratory technician). In these cases, survivors who have disclosed their experience to their social supports, including parents, friends, and extended family, are discouraged from reporting to authorities, as instances of sexual violence are frequently handled within communities or between the perpetrator and the family of the survivor. As such, survivors are often delayed in seeking care or do not receive the healthcare that they need. Additionally, many survivors may not disclose their experiences to their social supports at all, as they fear repercussions from their perpetrator or that they will not be believed. These survivors are “scared to voice out” because their social system may “feel that it’s a shame” (female nurse), “fear that they will be disgraced” (female nurse), or “say that it’s a lie” (female mental health provider).

***Structural barriers***. Providers discussed the role of structural barriers in impeding survivor’s access to post-assault healthcare. First, providers discussed how patients having to purchase medical supplies, evidence collection supplies, and STI and pregnancy prophylaxis is a barrier to receiving medical forensic services. One male doctor stated, “If you need to do a speculum exam, they have to buy it …You are buying a pregnancy kit and Hepatitis B/C screen …So essentially, they have to buy everything including your medication.” Survivors may be unable to receive needed treatment if required to purchase medical supplies, or may have to choose between supplies needed for a medical forensic exam and STI and pregnancy prophylaxis. One male doctor noted that patients sometimes purchase supplies and/or medications that they do not need or that end up not being used. These leftover supplies are kept on the medical unit and can be provided to survivors without funds to pay for treatment.

Providers also discussed how lack of privacy in the medical facility can function as a barrier to survivors seeking post-assault healthcare. Many providers reported that there is rarely private space available for conducting the examination, so patients are often examined in rooms or wards housing multiple patients. In these cases, screens or curtains are placed around the patient prior to the examination, yet providers acknowledged that patients may not feel comfortable disclosing sexual violence in this setting. As one female doctor stated, “Even then, a lot of the times they are not able to actually say what they want to say because, one, the hospital setting or environment is not even conducive. There are more than three people in the consulting room, there is no privacy for them, so they find it very difficult.” As such, many providers described the need for dedicated, private rooms to ensure that survivors have privacy.

Finally, many providers recognized that abuse and assault can also be experienced by males. A female nurse reported, “Males also do face it. They get and face gender-based violence.” Providers noted that although male survivors could seek healthcare from their institutions, many had never witnessed or heard of these cases where they work. Providers described a myriad of reasons male survivors do not report the violence or seek healthcare. First, stigma and fear may contribute to lack of reporting, in addition to a culture of silence around male survivorship. For example, providers discussed how stigma plays out for male survivors, in particular, as they may be confronted with rape myths from their community and healthcare providers. One male doctor stated, “It’s a whole societal stigma when a guy, sometimes even the attitude of healthcare workers towards them. ‘How can a woman rape you?’ And if it is a man too, there is a whole stigma about [being] gay. So, men are usually very shy to report such incidents.” Those male survivors that do seek healthcare are often faced with additional challenges. For instance, female survivors are sometimes sent to the antepartum unit to see a gynecologist, but providers recognized that there is no equivalent pathway for males. This sentiment is reflected by one male physician who said:

*The challenge would be that*, *because we don’t have a department that deals with rape*, *that will be outside our jurisdiction*, *because we work with just women*. *So probably they would have to get to the urology or probably the emergency physicians would have to work with them…We just deal with them because they are women and there is a form of gynecological challenge involved*. *Otherwise they would actually be lost*.

As such, male survivors may be discouraged from seeking healthcare following sexual violence, due to societal stigma and structural barriers.

### Triangulation of data

Results from the facility surveys, healthcare provider surveys, and healthcare provider semi-structured interviews all illustrate that hospitals in Ghana may have some of the basic structures and systems in place to provide care to survivors of sexual violence (e.g., HIV rapid testing, pregnancy testing, emergency contraception, analgesia, and antiemetics) but that sociocultural factors such as societal stigma and structural barriers are creating a culture where sexual assault survivors are unable or unwilling to access post-assault care. For example, the facility survey indicated that both hospitals had private rooms for examination of sexual assault survivors. However, during the semi-structured interviews providers discussed how lack of privacy in the medical facility can function as a barrier to survivors seeking post-assault healthcare. This dichotomy is illustrated in a quote from one female doctor, “Even then, a lot of the times they are not able to actually say what they want to say because, one, the hospital setting or environment is not even conducive. There are more than three people in the consulting room, there is no privacy for them, so they find it very difficult.” Additionally, the facility survey indicated that medications and supplies are available at both hospitals. However, only with the addition of the healthcare provider interviews did we learn that survivors of sexual violence must pay for these medications and supplies out of pocket. These costs serve as a barrier to many survivors receiving the comprehensive care they need. In sum, the combination of data obtained from the facility surveys, provider surveys, and provider interviews are necessary to understand the nuanced barriers and facilitators survivors of sexual violence experience when seeking post-assault care.

## Discussion and conclusions

In this mixed-methods situational analysis, 20 front-line healthcare providers in Cape Coast, Ghana completed surveys and participated in in-depth interviews related to the healthcare provider response to sexual violence. This response can be viewed as the entry point for enhanced services and responses for gender-based violence [28]. Using this framework, a survivor’s positive interaction with the healthcare facility could serve as the catalyst for a comprehensive rights-based response that addresses all of a survivor’s needs using a trauma-informed approach. However, in order to achieve this goal, there are some areas for improvement within these two hospitals.

The observational facility assessment and in-depth interviews with healthcare providers corroborated that there is a lack of supplies, knowledge, and training among healthcare providers. During the facility assessment, it became apparent that neither of the included sites had pre-packed rape kits available, which as one healthcare provider discussed, would have enabled them to better collect DNA and other evidence in the forensic examination. Additionally, neither site had PEP or informational handouts on medications (or side effects) and support services for survivors. This dearth of supplies and medications highlights the need for infrastructure to respond to this vulnerable patient population [3,8]. In addition to not having some necessary supplies, many of the providers reported a lack of education and training related to responding to survivors of sexual violence. Past studies have supported the need for healthcare provider training related to gender-based violence that considers sensitivity, confidentiality, privacy, and accountability [3,29–31]. At the institutional level, knowledge and training could be supplemented using formal protocols for responding to survivors of sexual violence and continued training supported by mentoring and supervision of new providers [3]. The findings of our study are consistent with the key outcomes that the Department for International Development has outlined when responding to violence against women and girls, including service delivery, capacity building, and an enabling environment [28]. All of these solutions highlight a need for an investment (socially and financially) in protecting and responding to survivors of gender-based violence [32].

Throughout the qualitative interviews, healthcare providers also described both personal and systemic barriers that inhibited survivors from reporting their assaults. Some of the personal barriers included stigma and, especially among male survivors, the belief that they cannot experience sexual violence. Systemic barriers included structural obstacles, such as the need to pay for supplies (including the “police form”) and medications required for a post-assault exam, as well as lack of privacy. In order to overcome the identified barriers, it is important for healthcare institutions to work with the community to develop resources [33]. This might include developing a Community Advisory Board, seeking support from Chiefs and leaders in the community, and working with the government to influence policy [32]. Past research in the maternal health arena has successfully utilized community mobilization principles to create interventions within communities that are acceptable, sustainable, and impact health outcomes [34,35]. This type of community buy-in and support at the local and national level is necessary to overcome victim-blaming and structural barriers that impede access to post-assault services.

Finally, participants described a lack of coordination in accessing resources. Based on the provider responses, it seemed that many survivors who reported to the police were compelled to present for a medical forensic exam to substantiate an assault and survivors who came to health centers first were encouraged to report to the police. However, there was limited discussion among our participants about other types of resources. Only one provider mentioned Ghana’s Domestic Violence & Victims Support Unit (DOVVSU), which is a part of the police force that specifically handles intimate partner and other gender-based violence cases. Furthermore, there are a lack of shelter services in Ghana for survivors to seek respite from abuse, despite specific language in the Ghana Domestic Violence Act of 2007 instructing that each district in the country should have a shelter [36]. The DOVVSU was in the process of constructing a shelter in Accra, which was abandoned due to a lack of funding [37]. Finally, mental health services in Ghana are limited by shortage of providers, stigma, and the geographic location of service provision [38]. In order to meet the comprehensive needs of sexual assault survivors, it is imperative that a coordinated, multi-sectoral response be established to provide resources beyond healthcare and law enforcement [32]. This requires health facilities to identify local resources and establish pathways that providers can use to facilitate timely referral of survivors [3].

### Limitations

This study is limited by its focus on one geographic region in coastal Ghana. Services for sexual assault are likely more available in larger urban areas of Ghana and even more limited in rural regions. Additionally, barriers to survivors accessing services are reported by healthcare providers. An important line of inquiry would be to interact with survivors themselves to assess the barriers they encountered. Furthermore, there is the potential for social desirability bias. The study team attempted to minimize this by using trained Ghanaian research assistants that were not a part of the healthcare system and by ensuring the confidentiality of all participants. Also, research assistants were students who were junior to all included healthcare providers, which may reduce this bias.

However, the strengths of this study include the use of a comprehensive observational facility assessment developed by an international organization focused on the prevention and response of sexual violence (SVRI) specifically to evaluate the status of services for sexual assault survivors [22]. Further, we used a mixed methods approach, surveying and interviewing front-line healthcare workers who are often the first professionals survivors of sexual assault encounter.

### Conclusions

This study provides a mixed-methods evaluation of the healthcare provider response to sexual violence in Ghana. The current healthcare response is limited by lack of supplies, knowledge, and training for healthcare providers. Further, healthcare providers described personal and structural barriers that may prevent survivors from accessing needed healthcare in the wake of sexual violence. Future work should focus on capacity building and multi-sectoral responses within local communities to ensure that the healthcare needs of survivors are met.
